# Recent Advances in Nutraceuticals for the Treatment of Sarcopenic Obesity

**DOI:** 10.3390/nu15173854

**Published:** 2023-09-04

**Authors:** Young-Chan Kim, Sang-Woo Ki, Hannah Kim, Sumin Kang, Hayoon Kim, Gwang-woong Go

**Affiliations:** Department of Food and Nutrition, Hanyang University, Seoul 04763, Republic of Korea; yck0220@gmail.com (Y.-C.K.); riddn3@naver.com (S.-W.K.); hkithaca00@hanyang.ac.kr (H.K.); sumin.kang708@gmail.com (S.K.); kimhayoon0408@gmail.com (H.K.)

**Keywords:** irisin, mitochondrial function, nutraceutical, sarcopenic obesity

## Abstract

Sarcopenic obesity, low muscle mass, and high body fat are growing health concerns in the aging population. This review highlights the need for standardized criteria and explores nutraceuticals as potential therapeutic agents. Sarcopenic obesity is associated with insulin resistance, inflammation, hormonal changes, and reduced physical activity. These factors lead to impaired muscle activity, intramuscular fat accumulation, and reduced protein synthesis, resulting in muscle catabolism and increased fat mass. Myostatin and irisin are myokines that regulate muscle synthesis and energy expenditure, respectively. Nutritional supplementation with vitamin D and calcium is recommended for increasing muscle mass and reducing body fat content. Testosterone therapy decreases fat mass and improves muscle strength. Vitamin K, specifically menaquinone-4 (MK-4), improves mitochondrial function and reduces muscle damage. Irisin is a hormone secreted during exercise that enhances oxidative metabolism, prevents insulin resistance and obesity, and improves bone quality. Low-glycemic-index diets and green cardamom are potential methods for managing sarcopenic obesity. In conclusion, along with exercise and dietary support, nutraceuticals, such as vitamin D, calcium, vitamin K, and natural agonists of irisin or testosterone, can serve as promising future therapeutic alternatives.

## 1. Introduction

As the world population ages, the incidence of both obesity and metabolic dysfunction increases, resulting in an increase in frailty and sarcopenia [1]. Elderly individuals are particularly susceptible to sarcopenia, a condition characterized by reduced muscle mass and strength. In 2022, the worldwide prevalence of sarcopenia was estimated to be between 10% and 27% in individuals aged 68.5 years [2]. Simultaneously, obesity rates have steadily increased from 1980 to 2019, with a worldwide prevalence of 14.0% in 2019. Compared to 1980, this was an increase of 9.4% [3]. The coexistence of sarcopenia and obesity, known as “sarcopenic obesity” (SO), is a relatively new concept that describes the simultaneous increase in fat mass and decrease in muscle mass in individuals [4]. Summarizing data from 50 studies updated in PubMed, Embase, and the Web of Science, the worldwide prevalence of SO has been reported to be 11% [5].

Sarcopenia is a term derived from the combination of the Greek words “sarx,” meaning flesh, and “penia,” meaning reduction [6]. Although there are no established criteria for diagnosing SO, it is associated with various adverse health outcomes, including physical disability, increased risk of falls, and poor quality of life [7]. Therefore, it is crucial to establish standardized criteria for identifying and treating SO. The lack of bioactive compounds and nutraceuticals that could be used to treat SO remains an issue. This review investigated the feasible diagnostic criteria, underlying causes, and viable management options for SO. Additionally, this article addresses ongoing research on the application of nutraceuticals as a potential therapeutic approach for SO.

## 2. Definition and Diagnosis

SO is diagnosed when muscle loss and fat mass gain co-occur. The definition of SO is based on the individual definitions of sarcopenia and obesity; however, an accurate definition has not yet been established. Although various definitions of sarcopenia have been proposed, a consensus has yet to be reached. Various diagnostic criteria for SO have been proposed by organizations and researchers [7]. To explore how SO is currently diagnosed, the definitions and diagnoses of sarcopenia and obesity are considered separately. Individuals meeting both the sarcopenia and obesity criteria were diagnosed with SO.

In a cross-sectional study conducted in 1998, the diagnostic criteria for sarcopenia in a sample of 883 elderly individuals were included in the New Mexico Elder Health Survey (NMEHS). They defined sarcopenia as a decrease in the relative skeletal muscle index and calculated as appendicular skeletal muscle mass (ASM). It was divided by height squared (ASM/height^2^) of two or more standard deviations below the reference values for young and healthy individuals and measured by dual X-ray absorptiometry (DXA) (Table 1) [8]. The skeletal muscle index (SMI = skeletal muscle mass/body mass × 100) was measured using bioelectrical impedance analysis to define sarcopenia in elderly Americans [9]. The Health ABC Study proposed an alternative definition of sarcopenia using appendicular lean mass (ALM). The authors also validated whether the ASM/height^2^ index could appropriately measure sarcopenia. Individuals with a high body mass index (BMI) and obesity had increased fat mass and reduced muscle mass. However, most patients had high absolute muscle mass and did not exhibit sarcopenia. Thus, it was confirmed that SO may be underestimated by the ASM/height^2^ index [10].

Sarcopenia is currently defined based on muscle mass, muscle strength, and anthropometric measures, including mid-arm and calf circumference [7]. The European Working Group for the Study of Sarcopenia (EWGSOP) defines sarcopenia as low muscle mass and function (strength or performance) and has developed an algorithm using gait speed (<0.8 m/s) before the measurement of muscle mass or strength [11]. The International Working Group for the Study of Sarcopenia (IWGS) provided a consensus on the definition. It is a combination of low appendicular lean mass and poor physical functioning (gait speed ≤ 1 m/s) [12]. The Foundation for the National Institutes of Health (FNIH) Sarcopenia Project proposed a causal and indirect relationship between muscle mass and function based on the definition of sarcopenia. It suggested the assessment of low lean muscle mass using DXA and reduced muscle function using handgrip strength [13]. The Asian Working Group for Sarcopenia suggested a diagnostic algorithm based on the available evidence in Asia. They followed the EWGSOP approach to define sarcopenia, which included handgrip strength and gait speed during the initial screening [14]. In 2018, the renewed EWGSOP2 was targeted to increase the early detection and treatment of sarcopenia. Low muscle strength was adopted as the main determinant of sarcopenia since muscle strength is regarded as a better criterion than muscle mass in predicting adverse consequences. The EWGSOP2 focuses on low muscle strength, using grip strength as the main parameter of sarcopenia. They used low muscle quantity and quality to confirm the diagnosis of sarcopenia and proposed measures of physical performance to assess its severity [15]. Although definitions of sarcopenia have been proposed, the lack of consensus requires further research to establish a standardized definition.

Obesity is a chronic disease in which the accumulation of excessive body fat leads to metabolic diseases and increased mortality. The World Health Organization (WHO) defines obesity as a BMI of over 30 kg/m^2^ or a waist circumference of a minimum of 102 cm for men and 88 cm for women. East Asia has a lower BMI cut-off point for the definition of obesity (25 kg/m^2^) [16]. Although BMI is a useful indicator of body fat, it is preferable to assess body fat distribution to predict the risk of metabolic syndrome and cardiovascular disease [17]. The American Association of Clinical Endocrinology recommends using the WHO body fat thresholds for the diagnosis of obesity, with a minimum of 25% in men and 35% in women [18].

**Table 1:** Diagnostic criteria of sarcopenic obesity.

Study	Sarcopenia Diagnosis Method	Measurement (Cut-Off Points)	Obesity Diagnosis Method (Cut-Off Points)
Baumgartner et al. [8]	ASM divided by height squared	DXA (men < 7.26 kg/m^2^; women < 5.45 kg/m^2^)	Body fat- men > 27%; women > 38%
Newman et al. [10]	ALM divided by height squared	DXA (men < 7.23 kg/m^2^; women < 5.67 kg/m^2^)	BMI ≥ 30 kg/m^2^
Cruz-Jentoft [11], EWGSOP	ASM divided by height squared	DXA (men ≤ 7.26 kg/m^2^; women ≤ 5.50 kg/m^2^) (Rosetta study) DXA (men ≤ 7.25 kg/m^2^; women ≤ 5.67 kg/m^2^) (Health ABC study) DXA (men ≤ 7.23 kg/m^2^; women ≤ 5.67 kg/m^2^) (Health ABC study) BIA (men: severe ≤ 8.50 kg/m^2^, moderate 8.51–10.75 kg/m^2^. women: severe ≤ 5.75 kg/m^2^; moderate 5.76–6.75 kg/m^2^) (NHANES III study)	NA
	Residuals	DXA (ALM (fat mass divided by height), men: −2.29; women: −1.73)	
	SMI divided by height squared	BIA (men ≤ 8.87 kg/m^2^; women ≤ 6.42 kg/m^2^)	
	Muscle strength	Handgrip strength (men < 30 kg; women < 20 kg)	
	Muscle strength based on BMI category	Handgrip strength (men: BMI ≤ 24: ≤ 29 kg, BMI 24.1–26: ≤ 30 kg BMI 26.1–28: ≤ 30 kg, BMI > 28: ≤ 32 kg; women: BMI ≤ 23: ≤ 17 kg, BMI 23.1–26: ≤ 17.3 kg BMI 26.1–29: ≤ 18 kg, BMI > 29: ≤ 21 kg)	
	Physical performance	SPPB (≤8-point score) Gait speed over 6 m (<1 m/s) (Health ABC study) Gait speed over 6 m (<1.175 m/s) (Health ABC study) Gait speed over 4 m (<0.8 m/s) (InCHIANTI study)	
LK Chen [14], AWGS	ASM divided by height squared	DXA (men < 7.0 kg/m^2^; women < 5.4 kg/m^2^) BIA (men < 7.0 kg/m^2^; women < 5.7 kg/m^2^)	NA
	Strength	Handgrip strength (men < 26 kg; women < 18 kg)	
	Physical performance	Gait speed over 6 m (< 0.8 m/s)	
Cruz-Jentoft [15], EWGSOP2	ASM	DXA/BIA (men < 20 kg; women < 15 kg)	NA
	ASM divided by height squared	DXA/BIA (men < 7.0 kg/m^2^; women < 5.5 kg/m^2^)	
	Strength	Grip Strength (men: < 27 kg; women < 16 kg) Chair stand > 15 s for five rises	
	Physical performance	Gait speed (≤ 0.8 m/s) SPPB (≤ 8-point score) TUG (≥ 20 s) 400 m walk test (non-completion or ≥ 6 min for completion)	

## 3. Pathophysiology and Complications

**Figure 1:**
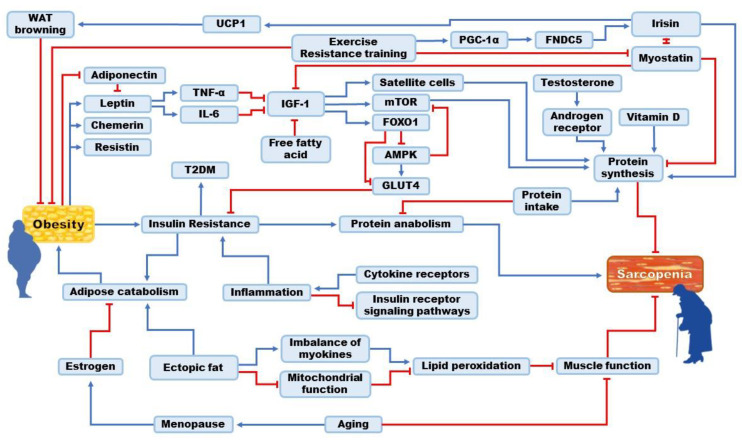
Mechanism of sarcopenic obesity (SO) and its association with endogenous metabolites.

Although the mechanisms and pathologies of SO are uncertain, multiple mechanisms have emerged as possible causes. Insulin resistance (IR), which is highly associated with type 2 diabetes mellitus (T2DM), serves as a connection between sarcopenia and obesity and frequently appears in aged and obese populations [19]. IR is stimulated by inflammatory molecules that directly intervene in the crosstalk between cytokines and insulin receptor signaling pathways [20,21]. Insulin is responsible for protein anabolism in muscle and adipose catabolism, and resistance to such signals inhibits protein synthesis while inducing proteolysis and adipose tissue growth (Figure 1) [19]. IR increases the size and number of adipocytes. Overall, the inhibition of protein anabolism and proneness to fat synthesis are consequences of insulin resistance and diabetes.

Another possible cause of SO is the inflammatory response and secretion of cytokines due to low-grade inflammation triggered by increased IR. Insulin resistance induces inflammation, and obesity activates macrophages, which leads to inflammation and the secretion of adipokines [22]. Inflammation in adipocytes leads to increased leptin, chemerin, and resistin levels and decreased adiponectin [6]. The increase in leptin induces upregulation of interleukin-6 (IL-6) and tumor necrosis factor-α (TNF-α) [21]. However, the effects of leptin are mediated by adiponectin [23]. Insulin-like growth factor-1 (IGF-1), responsible for the inhibition of autophagy of myocytes via Forkhead box O (FOXO) and mammalian target of rapamycin (mTOR), is suppressed by IL-6 and TNF-α [24,25]. IGF-1 promotes the proliferation of satellite cells, leading to muscle hypertrophy [26]. This decline prevents muscle growth and weakens muscle action. IGF-1 release is also inhibited by an increase in the concentration of free fatty acids [27]. Additionally, inflammation and oxidative stress also lead to the disposition of ectopic fat in muscles. Intramuscular fat accumulation causes mitochondrial impairment and an imbalance in myokines, inducing lipid peroxidation and impairing muscle action [6]. Similar to the outcomes of IR, inflammatory actions intermittently damage muscles and precipitate the accumulation of lipids, which are the two crucial causes of SO.

In addition, SO is related to sex hormones such as estrogen and testosterone. When women experience menopause, body weight and fat mass increase, especially in the abdominal region; however, a loss in lean mass is observed [28]. Inflammatory responses are stimulated during this period [29]. This promotes an increase in waist circumference and muscle reduction. These changes result from a decline in estrogen levels during menopause. In men, testosterone is responsible for muscle regeneration, protein synthesis through increased amino acid utilization, and the stimulation of androgen receptor expression [30]. Decreasing testosterone levels in men aged 65 years triggers muscle loss and reduces muscle strength and performance [21]. The reduction in testosterone and estrogen production in men and women provokes a decline in muscle mass while promoting fat production, ultimately resulting in SO.

As individuals age, there is a general decrease in food intake, particularly protein intake, which causes a decrease in muscle mass and strength. In addition to malnutrition, older adults tend to spend more time indoors than engaging in outdoor exercise. Muscle dystrophy occurs because of a lack of exercise, ultimately leading to more frequent indoor activity. Consequently, a decrease in the lean mass and an increase in the fat mass were observed. More time spent indoors leads to less exposure to the sun and, ultimately, reduced vitamin D [6]. Vitamin D deficiency causes type II muscle fiber atrophy, enlarged interfibrillar spaces, fat infiltration, and fibrosis [25]. Since vitamin D is responsible for protein synthesis, less exposure to sunlight inevitably leads to depressed muscle generation.

Myokine actions can also evoke SO, in addition to vitamin D, insulin, and inflammatory actions. Myostatin and irisin are currently being studied for their mechanisms and potential therapeutic approaches in muscle-wasting disorders, and it is expected that a better understanding of their mechanisms will contribute to the development of treatment and prevention strategies. Briefly, myostatin, the first discovered myokine, is expressed in the skeletal muscle to negatively regulate muscle synthesis. When myostatin is expressed, IGF-1, Akt, and mTOR are inhibited, and the FOXO1 pathway is stimulated to suppress glucose transporter 4 (GLUT4) and AMP-activated protein kinase (AMPK) [31,32]. Subsequently, the uptake of glucose by skeletal muscles is inhibited, leading to an increase in IR [33]. Myostatin is upregulated in obese humans and reduced after exercise. During physical activity, peroxisome proliferator-activated receptor γ coactivator-1α (PGC-1α) drives the expression of fibronectin type III domain-containing protein 5 (FNDC5), secreting irisin, the contradictory myokine to myostatin [34,35,36]. Further explanations of irisin are provided in a later section. The level of irisin decreases with age; alternatively, the level of myostatin increases and vice versa [37]. In summary, the secretion of myostatin not only causes muscle degradation but also induces the inhibition of irisin secretion, the myokine responsible for muscle production and fat reduction.

Sarcopenia and obesity are factors associated with poor quality of life, especially in adults. Functional capability, physical balance, and mortality are associated with muscle strength and body weight [38]. These effects are amplified when body size, specifically body weight, is substantial for poor muscle strength [20]. Higher muscle mass was positively correlated with greater physical performance. This means that when muscle mass declines, there is a greater risk of falls and fractures [39]. Osteoporosis and osteoarthritis are the two most common types of skeletal damage induced by SO. These are reciprocal procedures; thus, the occurrence of one event blocks another. Another study also compared the statistics of falls and fractures that occur in men and women. According to the New Mexico Elder Health Survey, compared by ALM/height^2^, men have a 2.5-fold greater likelihood of falling. However, no such association was observed among women [40]. Although there is no clear evidence, the rate of falls and fractures is expected to be related to visceral adipose tissue and ectopic fat [41]. Visceral fat and ectopic fat, which stimulate the pro-inflammatory system in the human body, are predicted to increase the likelihood of falls and fractures in obese individuals [42]. Patients diagnosed with SO often experience frequent falls and fractures, resulting in poor quality of life.

Increased body mass index (BMI), body weight, and reduced muscle mass are potential indicators of skeletal injury, including osteoarthritis and osteoporosis. Sarcopenia induces muscle weakness by loading more pressure on the hips and knees, two major body parts associated with osteoarthritis due to sarcopenia [43]. Additionally, increased body weight due to obesity places a greater burden on the lower limbs [44]. However, the pathological relationship between sarcopenia and osteoarthritis remains unclear [45]. Furthermore, the association between the two was independent of age, sex, and vitamin D levels [46]. Estrogen decline is a major regulator of bone metabolism and has been identified as a factor in SO [47]. Thus, a decline in estrogen levels and reduced skeletal strength lead to postmenopausal osteoporosis [48]. In summary, increased fat mass and decreased muscle mass among SO patients inevitably impose greater pressure and stress on the lower joints and bones, thereby contributing to the development of osteoarthritis and osteoporosis.

Additionally, SO is associated with increased mortality rates. A high waist circumference and low muscle strength are correlated with increased mortality [49]. Moreover, muscle strength and mass were independent of the mortality rate. A study conducted by Health, Aging, and Body Composition reported increased mortality resulting from less strength in the quadriceps [7]. Obese individuals were more prone to cancer, infection, steatosis, cirrhosis, etcetera [50]. The American Medical Association confirmed that mortality increased as the obesity grade increased. No association was observed between mortality and grade 1 obesity. However, aside from this grade, there was an increase in mortality in response to higher obesity levels [51]. A meta-analysis indicated that SO resulted in a higher mortality rate than in healthy individuals [49]. Low muscle strength and mass, along with obesity, expose individuals to a higher risk of various diseases such as cancer and cirrhosis, thereby elevating their proneness to mortality.

Sarcopenia and obesity are associated with metabolic disorders. Therefore, SO may have a greater impact on the metabolic syndrome (MetS) and may be associated with mortality rates, including T2DM, nonalcoholic fatty liver disease (NAFLD), dyslipidemia, hypertension, and cardiovascular disease (CVD), than sarcopenia or obesity alone [52]. The Korea National Health and Nutrition Examination Survey (KNHANES) evaluated sarcopenia, defined by muscle mass, with accompanying obesity (BMI ≥ 25 kg/m^2^). They reported that individuals with SO had an increased risk of dyslipidemia and a positive association with insulin resistance, as defined by Homeostatic Model Assessment (HOMA) scores and triglyceride levels [53]. A Korean SO Study (KSOS) cohort study reported that SO, defined by DXA-measured ALM/weight (%) and visceral fat, was associated with IR as assessed by the Homeostatic Model Assessment for Insulin Resistance (HOMA-IR) score, inflammation, C-reactive protein level, and vitamin D deficiency [54]. In summary, not only does SO increase mortality but also cause MetS in surviving individuals, depressing their quality of life.

## 4. Irisin and Its Role in Sarcopenic Obesity

### 4.1. Pathology and Implications of Irisin for Sarcopenia

**Figure 2:**
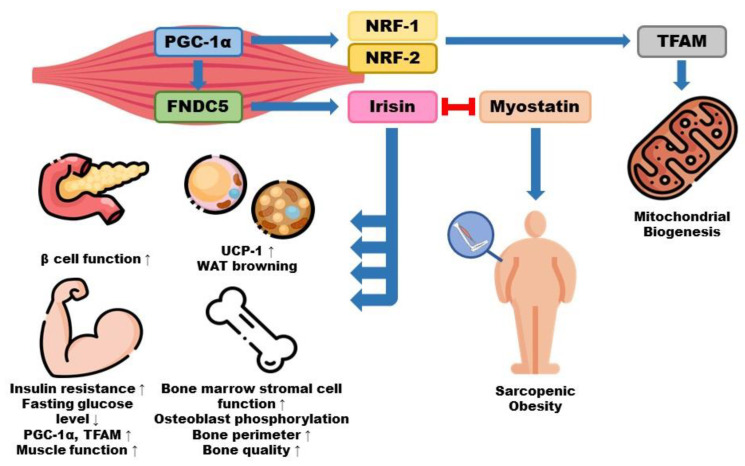
Mechanism of irisin and its intervention with myostatin and body components.

Irisin is secreted from muscles during exercise and inhibits myostatin secretion. Regular exercise promotes the secretion of FNDC5, a protein that induces irisin production [55]. Irisin, a myokine fragment of FNDC5, decreases fasting glucose levels and facilitates beta-cell function (Figure 2) [56]. In addition to regular exercise, irisin can be produced under environmental stresses such as caloric restriction, coldness, and oxidative stress [57]. Under these conditions, PGC-1α promotes nuclear respiratory factor-1 (NRF-1) and NRF-2 expression. Consequently, the mitochondrial transcription factor (TFAM) is promoted, resulting in mitochondrial biogenesis, fusion, and fission [58,59].

Although it is still unclear which type of exercise facilitates irisin secretion, multiple studies have verified that blood irisin levels increase instantly even after compulsory training, although the minimum increase is only 1.2-fold, depending on the intensity of the exercise (Table 2) [60,61]. Irisin secretion is not only a single-way procedure but also an interactive procedure, meaning that its secretion increases muscle biogenesis and is simultaneously increased by muscle growth [62]. In in vitro experiments, C2C12 myotubes treated with irisin showed increased TFAM and PGC-1α and significantly increased oxidative metabolism [59,63]. When applied to human myocytes and adipocytes, PGC-1α was upregulated, along with increased IGF-1 and decreased myostatin gene expression through the mitogen-activated protein kinase (MAPK) pathway. Simultaneously, muscle cell differentiation leads to increased mRNA expression of FNDC5 and irisin secretion [64]. Overall, the secretion of irisin and its precursors, FNDC5 and PGC-1α, is promoted under physical activities or other stressful conditions, and reciprocally, muscle growth also induces irisin production.

As mentioned earlier, other possible consequences of SO include osteoporosis and osteoarthritis. As muscle mass decreases, bone mass is reduced correspondingly [70]. To address this, irisin can be used to enhance bone marrow stromal cell differentiation and osteoblast phosphorylation. This prevents torsion, increases the bone perimeter, and enhances overall bone quality [63,71]. In addition, one of the major genes that deteriorates bone health is sclerostin, a major osteoblast differentiation-interrupting factor, via its conjunction with low-density lipoprotein (LDL) receptor protein 5/6 (LRP5/6) and frizzled coreceptors on the osteoblast surface to inhibit catenin signaling pathways [72]. However, not only is irisin eligible for reducing myostatin secretion, but serum sclerostin levels have also been reported to be inversely correlated with serum irisin levels [73]. In addition to promoting muscle prosperity, irisin mediates osteoporosis and osteoarthritis, which are possible outcomes of SO.

### 4.2. Pathophysiology and Implications of Irisin for Obesity

While inducing muscle hypertrophy, irisin can also induce an increase in uncoupling protein-1 (UCP-1) and energy expenditure in adipocytes, resulting in reduced lipid accumulation [73]. Serum irisin levels tended to be lower in patients diagnosed with obesity, independent of NAFLD, than in those that were not obese [74]. Moreover, although a positive correlation was observed between fat mass and myostatin levels, fat mass was negatively correlated with irisin levels [75]. Among the various treatments for obesity, exercise and physical activity are the most commonly recognized and effective solutions, along with caloric restriction [76,77]. Physical exercise promotes irisin secretion, eventually resulting in weight loss [78]. Irisin increases energy expenditure through increased mRNA expression of UCP-1 [79]. Consequently, triggered muscle shivering accelerates browning and thermogenesis regulation in white adipose tissue [24,80]. As a result, irisin can potentially treat various metabolic diseases such as T2DM, CVD, and NAFLD, which are well-known consequences of obesity.

In a study conducted in 2019, comprising two groups, a normal weight group and an obese group, a negative correlation was observed between irisin levels and BMI, waist circumference, fasting glucose level, insulin, HOMA-IR, Homeostatic Model Assessment of cell function (HOMA-B2), and the estimation of β-cell function in diabetic patients in the obese group [56]. In a similar experiment, obese patients with a BMI of an average of 36.9 kg/m^2^ were placed under irisin intervention, which improved their fat percentage and significantly reduced fasting insulin levels and HOMA-IR [65]. In in vivo studies, irisin was initially shown to be effective in browning white adipose tissue (WAT) in mice and treating obesity [63,66]. Fourteen to sixteen-week-old C57BL/6J mice were experimented with to determine whether swimming affected serum irisin levels. The group subjected to swimming as an exercise showed an increase in the levels of PGC-1α and FNDC5, which are stimulators of irisin secretion. Browning markers, including UCP-1, also increased in the group that swam [67]. Overall, obesity itself and its consequences are modified by irisin supplementation.

## 5. Approaches for the Treatment and Management

Although various attempts have been made to develop new drugs, there are currently no approved pharmacological treatments for SO [6]. Therefore, SO is managed through lifestyle therapies such as exercise, diet, and nutritional supplements. Bariatric surgery, targeted drug therapy, and advanced exercise are being actively researched for the treatment of SO [7]. Approaches involving nutraceuticals and dietary supplements have also been emphasized. Nutraceuticals with anti-inflammatory properties and potential improvements in mitochondrial function have been proposed as emerging treatment options for SO. Additionally, active research on nutraceuticals, such as irisin, as well as new dietary approaches, has demonstrated positive effects on both obesity and muscle loss [81].

### 5.1. Lifestyle Intervention

SO presents a unique challenge as it involves the interplay between sarcopenia and obesity. The recommended approach for treating SO involves lifestyle interventions aimed at reducing weight and fat mass while increasing muscle mass and strength, which may include modifications to diet, physical activity, or specific exercises. Exercise is a highly effective approach for managing cardiovascular and respiratory diseases, diabetes, and various cancers and has been proposed as a preventive and therapeutic strategy for obesity [82,83]. It can improve muscle mass and strength in older adults with sarcopenia [84]. Although the optimal exercise regimen has yet to be determined, combining resistance training and aerobic exercise is recommended [67]. Resistance exercise is considered an effective therapeutic strategy for treating muscle atrophy because it promotes muscle hypertrophy and muscle protein synthesis, thereby enhancing muscle strength, quality, and physical performance [85]. Aerobic exercise can improve not only fiber size and strength but also overall muscle size and strength [86]. Additionally, it increases mitochondrial biogenesis and mitophagy, enhancing mitochondrial turnover and protecting against sarcopenia [87,88]. Physical exercise boosts mitochondrial activity to enhance the muscle-to-fat ratio, and customized exercise programs are recommended for older adults.

To optimize SO management, it is crucial to broaden the scope beyond exercise alone and consider the importance of nutrition and functional ingredients. An important aim of nutritional supplementation in patients with SO is to increase muscle mass while reducing body weight, particularly fat mass [89]. A hypocaloric diet is an integral approach for reducing energy intake and facilitating weight loss and is ideally supplemented with vitamin D and minerals, particularly calcium [7]. Vitamin D is a vital micronutrient for fat and muscle metabolism [90]. It is critical to proper fat storage in the WAT, and its deficiency leads to ectopic fat deposition in other tissues, resulting in inflammation and insulin resistance [91].

Calcitriol, the active form of vitamin D, negatively affects parathyroid hormone (PTH) synthesis. PTH synthesis is inhibited when circulating calcitriol levels increase. Because PTH weakens muscle strength, vitamin D prevents the muscle from losing function [92,93]. According to a short-term longitudinal study conducted in 2020, a 65% increase in vitamin D was concealed 60 times after the vitamin D receptor (VDR) gene was expressed. In the same study, participants showed a significant decrease in total body fat (*p* = 0.001) and an increase in gynoid lean mass (*p* = 0.007) (Table 3) [94]. Furthermore, VDR overexpression is associated with skeletal muscle hypertrophy. In addition to muscle hypertrophy and skeletal muscle repair, vitamin D is reported to repair mitochondrial function and balance its activities to prevent oxidative stress, lipid peroxidation, and DNA damage [91,95]. Protection against IL-6-induced inflammation is a clinical effect of vitamin D [96]. Briefly, vitamin D can enhance muscle activity by improving mitochondrial activity and actuating defensive actions against inflammation.

Vitamin K, a fat-soluble protein-producing vitamin, is required for blood clotting and bone structure [97]. Although there is conflict regarding whether vitamin K can treat fractures and bone mineral density (BMD), vitamin K has the potential to increase serum vitamin concentration and produce bone resorption markers [7]. Menaquinone-4 (MK-4) or menatetrenone, one of the nine forms of vitamin K2, improves mitochondrial function by boosting mitochondrial electron transfer, resulting in more efficient ATP production [98]. Among all cell types in the body, skeletal muscle cells have the most abundant mitochondria. Mitochondrial imbalance weakens muscle strength and promotes muscle degeneration, ultimately leading to sarcopenia [99]. In an in vitro study, the release of lactate dehydrogenase (LDH), an enzyme used as a marker of muscle damage and sarcopenia, was reduced in muscle cells treated with MK-4 [98]. In a 2017 cohort study, menaquinone-7 (MK-7) was administered to postmenopausal women aged 55–65 to verify whether MK-7 affects body weight. In cells that responded well to osteocalcin carboxylation, MK-7 treatment significantly increased adiponectin levels and decreased abdominal fat mass and visceral adipose tissue [100]. Thus, vitamin K can deteriorate the factors that cause muscle damage and simultaneously reduce fat accumulation.

Among the essential minerals that counteract sarcopenia, including magnesium, selenium, and calcium, calcium plays the largest role in preventing and treating sarcopenia [101]. Calcium is necessary to maintain calcium kinetics and promote muscle contraction [102]. Muscle contraction is responsible for joint bending, stability, and posture, which are likely to decline in adults. Because of its myogenic regulation, calcium maintenance is important for retaining the contradictory actions of muscles [103]. The fourth KNHANES, conducted in 2009, revealed a relationship between calcium intake and muscle mass. Muscle mass decreases correspondingly with lower calcium intake [104]. Moreover, calcium and vitamin D play protective roles against sarcopenia and loss of muscle mass. Calcium intake also increases BMD, which repairs osteoporosis and osteoarthritis, which are consequences of SO [105]. A boost in muscle strength and bone mass are major calcium-induced consequences in the treatment of skeletal damage and muscle repair.

SO exerts greater pressure on the lower limbs and can ultimately trigger knee osteoporosis [106]. However, testosterone can be used to manage these problems. Decreased muscle mass and function due to testosterone loss can be reversed by testosterone therapy [107]. Testosterone therapy applied to older adults showed decreased fat mass in one study and improved hamstring and quadriceps strength in another [99]. Testosterone levels are also highly correlated with muscle function. This is a major factor in allowing the body to adapt to training and resistance exercise [30]. One study showed a dose-dependent increase in leg press strength when testosterone levels increased in older adults [89]. In addition to the functional aspects, muscle composition is affected by testosterone secretion [108]. Testosterone is likely to be responsible for muscle growth [109]. Secreted testosterone facilitates protein synthesis, which leads to muscle growth and increased muscle mass [110]. However, whether testosterone can be supplied through dietary action remains questionable. Although testosterone is not a component of food, some foods promote the production and secretion of testosterone [111]. Multiple diet types were tested to determine whether they affected the testosterone concentration. The NHANES, conducted in 2020, was designed to determine whether a plant-based diet index was associated with serum testosterone levels. It was concluded that these two factors are not associated [112]. Conversely, in another study in 2020, the ketogenic diet (KD) and Western diet (WD) were compared to determine which had a greater effect on testosterone levels. In KD, total testosterone significantly increased by 118 ng/dL but decreased by 36 ng/dL in WD [113]. Through these diets, an increase in testosterone levels strengthens the joints and muscles of the lower body and reduces the possibility of osteoporosis.

Fatty acids are proficient in regulating metabolism and body functions [114]. Many organizations, such as the WHO and Food and Agriculture Organization, have recommended polyunsaturated fatty acids (PUFA), especially n-3 PUFA [115]. Multiple fatty acids have been recognized for their ability to modify muscle and fat mass in the human body. Docosahexaenoic acid (DHA), eicosapentaenoic acid (EPA), and azelaic acid (AZA) are fatty acids that can be used to treat the distressing conditions of SO.

DHA, a ⍵-3 fatty acid commonly found in fish and milk, can delay skeletal muscle degradation related to obesity-induced inflammation [116]. When administered to C57BL/6J mice, DHA reduced inflammatory markers, and lipid accumulation was modified at the beginning of adipocytes [117]. The expression of genes related to mitochondrial biogenesis, PGC-1α, TFAM, and NRF-1, is also elevated in the presence of DHA in C2C12 muscle cells, proving DHA’s validity in both muscle genesis and lipid degradation [118]. DHA is adequate for generating proteins; however, it attenuates lipid accumulation by reducing inflammation.

EPA, a ⍵-3 fatty acid found in fish and nuts, can increase muscle function and reduce muscle damage [119]. When rats were treated with a 1 g/kg ratio, TNF-α and atrogin-1 levels were diminished, whereas myoblast determination protein (MyoD), a myogenic factor, increased [120]. In addition to boosting muscle, EPA is used to treat obesity. Mice fed a high-fat diet with EPA showed significantly reduced body weight, adiposity, adipocyte size, and macrophage infiltration into the adipose tissue. This group showed modifications in mitochondrial function [121]. Increased muscle and mitochondrial activity, along with improved adipocyte content, are outcomes that EPA is capable of bringing about.

By the same token, AZA, a ⍵-9 fatty acid mostly obtained from oatmeal and barley, can ameliorate muscle mass and fat capacity. During muscle hypertrophy, AZA promotes mitochondrial biogenesis and alleviates mitochondrial dysfunction. As a result, mitochondrial improvement reinforces muscle function and prevents muscle aging and contraction. Regarding obesity, AZA can attenuate fat mass as its consumption leads to a decline in lipid accumulation through the hydrolysis of triglycerides in adipose tissues. This disintegrates adipocytes, resulting in weight loss and body fat reduction [122]. Similar to the two fatty acids mentioned earlier, AZA can treat SO via mitochondrial biogenesis and adipose tissue collapse.

**Table 3:** The intervention of various nutrients on SO, muscle growth, and adipose tissue.

Reference	Intervention (Exposure, Dose, Duration)	Condition	Subjects (Sample Size, Gender, Age)		Markers
Medeiros et al. [94]	Cholecalciferol 2000 IU 60 days	Twins Not on vitamin supplementation	CG *n* = 45 Same sex Age: 18–45	SG *n* = 45 Same sex Age: 18–45	Total body fat ↓ Gynoid lean mass ↑ VDR mRNA expression ↑
Labudzynskyi et al. [96]	Vitamin D3 800 IU/kg 6 weeks	T1DM	Negative CG mice C56B1/J6 *n* = 8 21 ± 3 g	SG mice C56B1/J6 *n* = 8 21 ± 3 g	IL-6 mRNA expression ↓
Skinner et al. [107]	Testosterone replacement treatment	Hypogonadism	Placebo *n* = 1168 Age: ≥ 45	SG *n* = 1213 Age: ≥ 45	Fat free mass ↑ Total body strength ↑
Wilson et al. [113]	Ketogenic diet, western diet 10 weeks	-	*n* = 25 Age: college-aged		Serum testosterone level ↑ in KD
Rønning et al. [98]	MK-4 1 µM, 10 µM, 20 µM, 50 µM 6 days	Bovine primary skeletal muscle cells cultivated in 2% FBS with Ultroser G serum	CG *n* = 3000 cells/well	MK-4 10 µM *n* = 3000 cells/well	Cell proliferation ↑ Gap closure ↑
Knapen et al. [100]	MK-7 180 mcg/day 3 years	Postmenopausal women	SG *n* = 107 Female Age: 55–65	Placebo *n* = 107 Female Age: 55–65	Adiponectin ↑ Abdominal fat ↓ Visceral adipose tissue area ↓
Félix-Soriano et al. [117]	DHA fish oil 15% HFD 3 times/day 12 months	Fed HFD for 4 months	HFD mice *n* = 10 Age: 6 months	HFD + DHA mice *n* = 6 Age: 6 months	Visceral white adipose tissue ↓ Subcutaneous white adipose tissue ↓ Body weight ↓ TFAM ↑ Beige adipose tissue markers ↑
LeMieux et al. [121]	EPA 10% HFD 11 weeks	-	HFD mice *n* = 8–10	HFD + EPA mice *n* = 8–10	Body weight ↓ Adiposity ↓ Adipocyte size ↓
Osella et al. [66]	Low glycemic index diet 6 months	Metabolic syndrome	CG *n* = 80	SG *n* = 55	Serum irisin concentration ↑ Fat free mass ↑
Estell et al. [71]	Irisin 2–20 ng/mL 4 h–7 days	Osteoblasts	CG	SG	Osteoblasts/well ↑
Zafar et al. [69]	Low glycemic index diet ≥1 week	Insulin resistance, T1DM, T2DM	*n* = 54		Glycated hemoglobin ↓ Fasting glucose ↓ BMI ↓ Total cholesterol ↓ LDL ↓

### 5.2. Emerging Therapy

#### 5.2.1. Efficacy of Nutraceuticals

Polyphenols with anti-inflammatory and antioxidant properties have been proposed as potential nutraceuticals for treating sarcopenia by enhancing mitochondrial function [123]. Oligonol, a low-molecular-weight polyphenol derived from lychee, possesses anti-inflammatory and anti-obesity properties [124,125]. Recent studies have shown that oligonol regulates protein degradation related to mitochondrial function and improves skeletal muscle quality [126,127]. In a study conducted using an ovariectomized rat model, oligonol supplementation revealed the molecular mechanisms underlying its effects on body composition, protein turnover, and mitochondrial quality [128]. In this study, significant changes in body weight and accumulated fat were observed without any muscle loss. Oligonol treatment decreases sterol regulatory element-binding protein 1 expression. In another study, oligonol supplementation was shown to regulate body weight and improve serum lipid profiles in obese women [129]. Oligonol was found to increase AMPKα activity and enhance peroxisome proliferator-activator receptor α expression, thereby inhibiting lipid accumulation within the muscle [126]. Oligonol activates phospho-mTOR and its related pathways and stimulates the expression of FOXO and MuRF1, thereby increasing protein turnover. Oligonol also influences the expression of PGC-1α and NRF2, which are associated with mitochondrial biogenesis. Oligonol has the potential to serve as a treatment for SO as it inhibits lipid accumulation without muscle loss and regulates protein turnover and mitochondrial function [130].

Curcumin, also known as diferuloylmethane, is a major polyphenolic component that imparts a yellow color to turmeric [128]. The health benefits of turmeric have been attributed to curcumin. Curcumin possesses anticancer, antioxidant, anti-inflammatory, antidepressant, and anti-aging properties. Curcumin has been reported to have beneficial effects on neurological disorders, neuromuscular diseases, and osteoarthritis [131]. Several studies have revealed that impaired expression of nuclear factor erythroid-2 related factor-2 (NFE2LE) during aging is associated with oxidative stress and muscle degeneration [132]. Curcumin also activates NFE2LE [133]. It has been demonstrated that curcumin increases the expression and activation of NFE2LE by promoting the release of NFE2LE from kelch-like ECH-associated protein 1 [134]. Upregulation of NFE2LE in the skeletal muscle is induced by curcumin supplementation [135]. In one experiment, long-term administration of curcumin was conducted in rats, followed by evaluation of muscle mass and function. When 32-month-old male F344xBN rats were fed a diet containing 0.2% curcumin for 4 months, they exhibited greater plantaris mass and increased force production compared to the control group. Additionally, rats that consume curcumin have higher nuclear fraction levels of NFE2LE and lower oxidative macromolecular damage [136]. In summary, curcumin has the potential to be a therapeutic agent for SO owing to its anti-aging effects, such as antioxidant and anti-inflammatory properties, and its ability to activate NFE2L2.

Together, green tea extract (GTE) and other substances can treat sarcopenia and obesity. GTE facilitates muscle function and induces weight loss. GTE not only suppresses muscle loss and improves muscle function but can also increase satellite cell proliferation and stimulate differentiation of the plantaris and soleus muscles after hindlimb suspension [137]. Similarly, 15-day supplementation with 500 mg GTE daily reduced muscle damage markers after exercise, leading to muscle recovery [138]. In addition to enhancing muscle structure, GTE plays an important role in weight management. After endurance training, a group supplemented with GTE exhibited increased serum irisin concentrations and significant reductions in body weight, BMI, body fat percentage, and visceral fat [139]. Moreover, when GTE was administered to obese women with a BMI > 27 kg/m^2^ and a weight circumference of 80 cm or above for 12 weeks, a consistent decrease in total cholesterol and LDL plasma levels was observed with no side effects. These results suggest that they may be associated with the inhibition of ghrelin secretion, leading to increased adiponectin levels [140]. Ultimately, increased serum irisin levels and muscle mass and decreased markers of obesity were observed after supplementation with GTE.

Epigallocatechin gallate (EGCG), commonly found in green tea, is known for its ability to translocate GLUT4 and reinforce muscle function. Not only does GLUT4 translocation fix IR, but it also boosts glycogen storage in muscles, sustaining muscle performance [141]. ECGC also acts as an antioxidant by reversing the increase in reactive oxygen species. Such antioxidant actions, GLUT4 translocation, and activation of the AMPK pathway regulate glucose metabolism in skeletal muscles and diminish fatty acid synthesis, reducing body weight during muscle development [142]. In an experiment supplementing sarcopenic rats with 200 mg/kg body weight ECGC, the expression of anabolic factors, such as IGF-1, increased significantly, and the muscle was preserved [143]. Myogenin and MyoD levels were increased, whereas myostatin levels declined when aged mice were supplemented with ECGC. Moreover, the RNA expression of mitochondrial metabolism-related molecules, such as mitochondrial cytochrome b and mitochondrial cytochrome c oxidase II, III, and IV, increased when old mice were treated with ECGC along with treadmill runs for 10 weeks [144]. Catechin and epicatechin are flavonols found in green tea extract [145]. These two myostatin inhibitors have pharmacotherapeutic effects against SO [146]. When two groups of males were treated with epicatechin or a placebo after resistance exercise for 8 weeks, the group supplemented with epicatechin showed significantly greater myostatin inhibition than the placebo group (*p* ≤ 0.05) [147]. Overall, EGCG, catechin, and epicatechin were the bioactive compounds in GTE that demonstrated a superior effect in the treatment of SO.

Green cardamom, a perennial plant in the ginger family native to southern India, has attracted attention as a potential treatment for SO. Green cardamom has therapeutic effects on MetS, including diabetes, hyperlipidemia, obesity, and high blood pressure [148]. Green cardamom improves several blood factors such as lipids, inflammatory markers, liver enzymes, and irisin (Table 4) [149]. This study was conducted at the National Iranian Oil Company Central Hospital in Tehran to test the effect of green cardamom on irisin secretion. Subjects supplemented with three capsules of 500 mg green cardamom daily for 3 months showed a significant increase in serum irisin, HDLc, quantitative insulin sensitivity check index, and decreased fasting blood insulin level, triglycerides, LDLc, HOMA-IR, and fatty liver grade (*p* < 0.05) [150]. Green cardamom can prevent MetS-related obesity and enhance irisin production, thereby potentially treating SO.

A-terpineol (TPN), an alcoholic compound found in essential oils extracted from green cardamom, reduces inflammation and insulin resistance [151]. Mice treated with different concentrations of TPN (25, 50, and 100 mg/kg) showed decreased inflammatory responses and diseases caused by TNF-α and prostaglandin E₂ [152]. In another study, mice fed a high-fat diet showed counteractions against insulin resistance when treated with 50 mg of TPN. The same study evaluated whether TPN can potentially reduce proinflammatory cytokines, such as IL-6 and TNF-α, that lead to obesity [153]. Linalool, another compound in green cardamom, reduces hyperalgesia and non-inflammatory muscle pain [154]. Limonene, a cyclic monoterpene found in green cardamom, stimulates osteoblast differentiation and nullifies the effects of p38 inhibitors. P38 is a type of MAPK responsible for osteoblast differentiation and muscle regulation; thus, when inhibited, it prevents muscle recovery [155]. Limonene also acts as an antioxidant, reducing lipid peroxidation and superoxide dismutase levels after muscle injury [156]. In summary, TPN and limonene, the major nutraceuticals in green cardamom, inhibit inflammatory cytokines by treating obesity and facilitating muscle recovery.

Garlic is a commonly used spice well known as an effective antioxidant that simultaneously diminishes bacteria and parasites and lowers blood pressure and cholesterol levels [157]. In a study in which subjects were administered two tablets consisting of 400 mg of garlic powder, a significant decrease in body weight and fat mass was observed compared with the group that received placebo tablets (*p* < 0.05) [158]. In another study, subjects with NAFLD were supplemented with four tablets of 400 mg garlic powder and compared with the control group. A significant reduction in waist circumference (*p* = 0.001), body fat percentage (*p* < 0.001), fasting glucose level (*p* = 0.01), and insulin resistance (*p* < 0.001) were observed, along with a significant increase in skeletal muscle mass (*p* = 0.002) [159]. Garlic has the ability not only to increase muscle mass but also to lower blood pressure, cholesterol levels, and obesity.

To explore the compounds in garlic that contribute to the therapeutic effects of SO, s-allyl cysteine (SAC), a compound regularly found in garlic, has been highlighted as a potential defender against muscle atrophy [160]. C2C12 myotubes were exposed to 100 µM H₂O₂ and supplemented with 400 µM SAC to examine whether SAC exerted an anti-atrophic effect. SAC reduces myostatin secretion and mediates the degradation of muscle-specific proteins, proving that it is an effective anti-atrophic compound [161]. In another experiment, C2C12 was treated with 100 ng/mL TNF-α. Groups present in SAC and without SAC were compared to detect that the group supplemented with SAC restricted TNF-α-induced proteolysis and protected myotubes against inflammatory molecules, such as IL-6 and TNF-like weak inducers of apoptosis [162]. SAC protects garlic against muscle atrophy by inhibiting myostatin production and diminishing inflammatory responses.

**Table 4:** Studies of potential nutraceuticals and their results related to SO.

Reference	Substance (Compound)			Intervention (Exposure, Dose, Duration)	Condition	Subjects (Sample Size, Gender, Mean Age)		Markers
Daneshi-Maskooni et al. [149]	Green Cardamom			2 cardamom 500 mg capsules 3 times/day 3 months	Overweight or obese, NAFLD	Placebo *n* = 44 Age: 30–60	Supplement group *n* = 43 Age: 30–60	TNF-α ↓ IL-6 ↓ ALT ↓ Degree of fatty liver ↓
Daneshi-Maskooni et al. [150]				2 cardamom 500 mg capsules 3 times/day 3 months	Overweight or obese, NAFLD	Placebo *n* = 44 Age: 30–60	Supplement group *n* = 43 Age: 30–60	Irisin ↑ HDL-c ↑ TG ↓ LDL-c ↓ HOMA-IR ↓ Degree of fatty liver ↓
Oliveira et al. [152]	Green Cardamom		α-terpineol	α-terpineol 25, 50, 100 mg/kg 180 min	Hypernoinception induced by carrageenan	Male Swiss mice *n* = 6 per group Age: 2–3 months Mass: 28–32 g		TNF-α ↓ Prostaglandin E₂ ↓
Sousa et al. [153]				A-terpineol 25, 50, 100 mg/kg 6 weeks	Obese, high-fat and hypercaloric diet	Sqrague-Dawley rats *n* = 6 per group Age: 21 days Mass: ≃150 g		TNF-α ↓ IL-1β ↓ Weight gain ↓ Degree of fatty liver ↓
Nascimento et al. [154]			Linalool	Linalool 25 mg/kg Alternate days for 27 days	Hyperalgesia, injected with 20 μL pH 4 acidic saline	Male Swiss mice Mass: 25–30 g		Non-inflammatory pain ↓
Soundharrajan et al. [155]			Limonene	Limonene 2.5, 5, 10 μM 6 days	Seeded 5 × 10⁴ cells/well, 10% FBS in DMEM, 37 °C, 5% CO₂	C2C12 skeletal cell 80–90% confluency		Calcium deposition ↑ Myogenin ↑ MyoD ↑ p38 MAPK signalling pathway ↑
Santos et al. [156]				Limonene 5% 96 h	Gastrocnemius muscle injured by 0.459 kg metal bar press with 0.811 J	Male Wistar rats		Thiobarbituric acid reactive substances ↓ Superoxide dimutase ↓
						Muslce injured group *n* = 6 Mass: 250–280 g	Supplemented group *n* = 6 Mass: 250–280 g	
Silva et al. [138]	Green Tea			GTE 500 mg/day 15 days	Exerecise-induced muscle soreness	Placebo *n* = 10	Supplemented group *n* = 10	Muscle recovery ↑
Bagheri et al. [139]				GTE 500 mg/day 8 weeks	Under endurance training: Circuit training, fast walking, jogging 3 times/week	Placebo *n* = 15	Supplemented group *n* = 15	IL-6 ↓ Adiponectin ↑ Irisin ↑ Body weight ↓ BMI ↓ Body fat percentage ↓ Visceral fat area ↓
Chen et al. [140]	Green Tea	EGCG		EGCG 856.8 mg/day 12 weeks	BMI ≥ 27 kg/m^2^, waist circumference ≥ 80 cm	Placebo *n* = 39 Age: 20–60	Supplemented group *n* = 38 Age: 20–60	Body weight ↓ BMI ↓ Waist circumference ↓ TC ↓ LDL-c ↓ Ghrelin ↓ Adiponectin ↑
Meador et al. [143]				EGCG 200 mg/kg 8 weeks	Sarcopenia	Sprague-Dawley rats		IGF-1 ↑
						Control group Age: 20 months	Supplemented group Age: 20 months	
Mafi et al. [147]		Epicatechin		Epicatechin along with resistance training	Sarcopenia	Placebo *n* = 15 Age: 68.63 ± 2.86	Supplemented group *n* = 15 Age: 68.63 ± 2.86	Myostain ↓
Soleimani et al. [158]	Garlic			2 garlic powder 400 mg tablets/day 15 weeks	NAFLD	Placebo *n* = 45 Age: 20–79	Supplemented group *n* = 45 Age: 20–70	Body weight ↓ Body fat mass ↓
Sangouni et al. [159]	Garlic			4 garlic powder 400 mg tablets/day 12 weeks	NAFLD	Control group *n* = 43 Age: ≥ 18	Supplemented group *n* = 45 Age: ≥ 18	Waist circumference ↓ Body fat percent ↓ Fasting glucose level ↓ Insulin resistance ↓ Skeleta muscle mass ↑
Gupta et al. [161]	Garlic	SAC		SAC 200 μM 48 h	Atrophic effect by H₂O₂ DMEM, FBS, 2% horse serum, 1 μg/mL ciprofloxacin, 1.25 μg/mL amphotericin B	C2C12 muscle cell 80–90% confluency		TWEAK ↓ IL-6 ↓ Myostatin ↓ Muscle denervation ↓
Dutt et al. [162]				SAC 0.01 mM 72 h	Treated with TNF-α 100 ng/mL Seeded 2 × 10⁶ cells/well, DMEM with 20% FBS, 5 μg/mL ciprofloxacin, 2.5 μg/mL amphotericin B, 37 °C, 5% CO₂	C2C12 muscle cell 70–80% confluency		TNF-α ↓ IL-6 ↓ IL-1β ↓ TWEAK ↓
Chang et al. [127]	Oligonol			200 mg/kg oligonol 8 weeks	Senescence-accelerated	SAMP8 Mice		phosphorylation of AKT/mTOR/p70sk6 ↑ MuRF-1/MAFbx ↓ PGC-1α/Tfam ↓ Mfn2/Opa1 ↓ cytochrome c ↓
Receno et al. [136]	Curcumin			0.2% curcumin 4 months	Aged	F344xBN rats ad libitum control (CON; *n* = 18) 0.2% curcumin (CUR; *n* = 18) pair-fed (PAIR; *n* = 18) Age: 32 months		Food intake ↓ plantaris mass ↑ force production ↑ nuclear fraction levels of Nrf2 ↑ oxidative macromolecule damage ↓

#### 5.2.2. Dietary Interventions to Modulate Irisin Secretion

In addition to lifestyle interventions, multiple dietary methods can also enhance irisin production. Downregulation of FNDC5 was observed when mice were fed a high-fat diet, whereas a high-carbohydrate and high-protein diet showed the opposite result. A high-protein diet maintained FNDC5 and irisin levels and increased brown adipose tissue in mice (Table 2) [163]. Although controversial, some studies have reported that meat consumption is negatively correlated with blood irisin levels [68]. However, increased intake of vegetables, cheese, and processed meat effectively increases irisin secretion [66].

The most common form of diet that manages SO is a low-glycemic-index diet (LGID). Several studies have been conducted to determine whether LGID effectively improves irisin levels. In one study of subjects with MetS, the LGID was compared with the Mediterranean diet (MD) and low-glycemic index Mediterranean diet (LGIMD). Subjects with LGID showed higher irisin levels than those with MD and LGIMD [66]. In another study, LGID led to weight loss; however, a greater reduction was observed in those with normal blood glucose levels than in those with higher blood glucose levels. Body fat was significantly reduced in those with normal glucose tolerance, but those with impaired glucose tolerance, or T2DM, did not show a decrease. In addition, LGID lowers fasting glucose and glycated hemoglobin levels. LGID was also able to reduce BMI, total cholesterol (TC), and low-density lipoprotein cholesterol (LDLc), but did not enhance fasting insulin or high-density lipoprotein cholesterol (HDLc) [69,164]. In addition to a high-protein diet and increased vegetable intake, LGID mice showed increased irisin secretion and reduced levels of obesity markers such as fasting glucose and cholesterol.

## 6. Conclusions

Aging populations have emerged as a social issue in many countries, increasing the prevalence of sarcopenia due to the age-related decline in muscle mass and strength. Additionally, there is a simultaneous increase in the prevalence of SO when sarcopenia coexists with obesity. SO can have adverse consequences such as physical disability, poor quality of life, metabolic syndrome, and mortality, negatively affecting individuals and society. Although the definition and pathology of SO have continued to develop and be suggested, more research is essential to obtain a clear pathology and a consensus definition. Various treatments have been suggested for SO. The most important factors are increased skeletal muscle mass and decreased body fat mass. Therefore, exercise has been proposed as a fundamental treatment. However, implementing an optimal diet is also important for maximizing recovery from SO. As mentioned in this paper, several dietary methods can have positive effects, such as calcium supplementation, vitamins D and K, a ketogenic diet, and a high-protein diet. Moreover, irisin, a myokine released during exercise or under specific stressful conditions, is competent in ameliorating muscle and fat content while canceling the secretion of myostatin, a myokine that reverses irisin. Nutraceuticals such as green cardamom, which can inhibit myostatin secretion, promote irisin secretion, or treat obesity, may also be a possible aid in managing SO. Although the treatment of SO through dietary effects and exercise remains questionable, attempts to treat it using nutraceuticals offer a potential therapeutic approach by targeting various mechanisms of action.

## Figures and Tables

**Table 2:** Interaction between irisin and sarcopenic or obese patients and causes and potential consequences of irisin secretion.

Reference	Intervention (Exposure, Dose, Duration, Follow-Up)	Condition	Subjects (Sample Size, Gender, Age)				Results
Norheim et al. [60]	Endurance and strength training 12 weeks	Inactive, hyperglycaemic, overweight, pre-diabetic	Normal weight *n* = 13 Age: 40–65		Obese group *n* = 13 Age: 40–65		Irisin ↑ PGC-1α ↑ FNDC5 ↑
Tsuchiya et al. [61]	High-intensity exercise 40 min	Healthy, sedentary	*n* = 6 Age: 22.5 ± 1.1 Height: 174.8 ± 2.8 cm Weight: 67.1 ± 2.2 kg BMI: 22.1 ± 1.1				Irisin ↑ LDH ↓
Vaughan et al. [59]	Irisin 5 nM 24 h	5 × 10⁵ cells/well, DMEM containing 4500 mg/L glucose, 10% FBS, 100 U/mL penicillin/streptomycin, 37 °C, 5% CO₂	C2C12 muscle cell				Mitochondrial content ↑ NRF-1 ↑ TFAM ↑ GLUT4 ↑ UCP-3 ↑
Huh et al., (2014) [64]	Irisin 10 nM, 50 nM 12 days	Muscle collected from healthy subjects	3T3L1 adipocyte				Irisin ↑ FNDC5 ↑ IGF-1 ↑ PGC-1α ↑ UCP-1 ↑
Rashid et al. [56]	Moderate exercise 6 months	Obesity	Normal group *n* = 30 Age: 20–43 BMI: <25 kg/m^2^		Obese group *n* = 30 Age: 20–43 BMI: ≥30 kg/m^2^		Irisin ↑ BMI ↓ Waist circumference ↓ Fasting glucose level ↓ Fasting insulin level ↓ HOMA-IR ↓ HOMA-B2 ↓
Fukushima et al. [65]	Diet, exercise, behavioral therapy 6 months	Obesity	*n* = 22 5 males, 17 females Age: 46.1 ± 16.0 BMI: 36.9 ± 5.0 kg/m^2^				Irisin ↑ BMI ↓ Body fat percentage ↓ Subcutaneous fat area ↓ Triglycerides ↓ HOMA-IR ↓ Fasting glucose level ↓
Osella et al. [66]	Low glycaemic index diet, Mediterranean diet, Low glycaemic index Mediterranean diet 24 weeks	Metabolic syndrome	Control group *n* = 80	LGID group *n* = 55	MD group *n* = 51	LGIMD group *n* = 45	Irisin ↑ Saturated fatty acids ↓ Fat free mass ↑ BMI ↓
Cho et al. [67]	Swimming 90 min	Acclimitized to swimming	C57BL/6J				FNDC5 ↑
			Control group *n* = 10 Age: 14–16 weeks		Swimming exercise group *n* = 10 Age: 14–16 weeks		
Mâcedo et al. [68]	High protein diet 60 days	-	Standard diet mice *n* = 7		High protein diet mice *n* = 7		Brown adipose tissue ↑
Zafar et al. [69]	Low glycemic index diet ≥1 week	Insulin resistance, T1DM, T2DM	*n* = 54				Glycated hemoglobin ↓ Fasting glucose ↓ BMI ↓ Total cholesterol ↓ LDL ↓

## Data Availability

The data presented in this study are available within the article.
